# Smoke-Free Policies and 30-Day Mortality Rates for Chronic Obstructive Pulmonary Disease

**DOI:** 10.34172/ijhpm.2021.74

**Published:** 2021-07-14

**Authors:** Hanadi Hamadi, Sericea Stallings-Smith, Emma Apatu, Breck Peterson, Aaron Spaulding

**Affiliations:** ^1^University of North Florida, Jacksonville, FL, USA.; ^2^McMaster University, Hamilton, ON, Canada.; ^3^Mayo Clinic, Jacksonville, FL, USA.

**Keywords:** Smoke-free Policies, COPD, 30-Day, Mortality, Secondhand, Obstructive Pulmonary Disease

## Abstract

**Background:** Smoke-free policies have been shown to impact 30-day readmission rates due to chronic obstructive pulmonary disease (COPD) among adults aged ≥65 years. However, little is known about the association between smokefree policies and 30-day mortality rates for COPD. Therefore, we investigated the association between comprehensive smoke-free policies and 30-day mortality rates for COPD.

**Methods:** We used a cross-sectional study design and retrospectively examined risk-adjusted 30-day mortality rates for COPD across US hospitals in 1171 counties. Data were sourced from Centers for Medicare and Medicaid Services (CMS) Hospital Value-Based Purchasing (HVBP) Program, American Hospital Association (AHA) Annual Surveys, US Census Bureau Current Population Survey, and US Tobacco Control Laws Database from the American Nonsmokers’ Rights Foundation (ANRF). Data were averaged at the county level for years 2015-2018. Hierarchical Poisson models adjusted for differences in hospital characteristics and accounted for the clustering of hospitals within a county were used.

**Results:** Our findings show a consistent association between stronger smoke-free policies and a reduction in COPD mortality. When evaluating smoke-free policy, county characteristics, and hospital characteristics individually, we found that counties with full coverage or partial coverage had a reduced incidence rate of COPD mortality compared to no coverage counties. After adjusting for the county and hospital characteristics, counties with full coverage of smoke-free policies had a reduced rate of 30-day COPD mortality (adjusted incidence rate ratio [IRR]: 0.87, 95% CI: 0.79, 0.96) compared to counties with no policy coverage.

**Conclusion:** Comprehensive smoke-free policies are associated with a reduction in 30-day mortality following hospital admission for COPD. Partial smoke-free legislation is an insufficient preventative measure. These findings have strong implications for hospital policy-makers, suggesting that policy interventions to reduce COPD-related 30-day mortality should include implementing smoke-free policies and public health policy-makers to incentivize comprehensive smokefree policies.

## Introduction

**Key Messages**

** Implications for policy makers**

- Comprehensive smoke-free policies are associated with reducing 30-day in-hospital chronic obstructive pulmonary disease (COPD) mortality.
- Partial smoke-free legislation is an insufficient preventative measure.
- Policy interventions that are known to reduce poverty and improve continuity of hospital care in rural areas should be considered in conjunction with smoke-free policies.

** Implications for the public**

Secondhand smoke exposure is a risk factor for chronic obstructive pulmonary disease (COPD) development, exacerbations, and mortality. Comprehensive smoke-free policies that prohibit smoking entirely in public spaces are important public health countermeasures that minimize secondhand smoke exposure. Partial policies are less protective in reducing secondhand smoke exposure. Our findings indicate that comprehensive smoke-free policies are associated with saving lives. In addition, counties with a greater percentage of Medicare and Medicaid coverage had a reduced risk of COPD mortality. Counties and states should prioritize implementing smoke-free policies and structural interventions that increase access to affordable healthcare, which benefits existing public health programs, hospitals, and patients.

Chronic obstructive pulmonary disease (COPD) is a slowly progressive, non-reversible lung disease that includes chronic bronchitis, emphysema, and refractory asthma.^1^ A confirmed diagnosis of COPD is when airflow obstruction FEV1/FVC ratio is less than 0.70.^2^ COPD affects an individual’s physical, social, and mental well-being, and results in an increased incidence of comorbidities such as cardiovascular disease, diabetes, mental disorders, and physical disabilities.^3-5^ In the United States, COPD affects 6.2% (age-adjusted) of the adult population and is the third leading cause of death.^6,7^ Prior research shows that COPD has a direct national economic burden that continues to increase annually and was estimated to reach $49 billion by the end of 2020.^8-12^ Therefore, the Centers for Medicare and Medicaid Services (CMS) began using 30-day mortality COPD as a marker of hospital performance and a driver for hospital reimbursement.^13^

 While other risk factors, including indoor air pollution, occupational exposures, and genetics play a role in the development of COPD, cigarette smoking contributes to nearly 80% of COPD mortality.^8,14^ A meta-analysis of 129 studies determined a causal relationship among persons who ever smoked and COPD.^15^ Secondhand smoke exposure is also a relevant risk factor for COPD development, exacerbations, and mortality. For example, a study has observed an association between secondhand smoke exposure in childhood and increased COPD mortality (hazard ratio = 1.31; 95% CI: 1.05-1.65) among never smokers.^16^ Another study identified similar COPD prevalence among active and non-smokers within the same geographic area, suggesting a relationship between regions, secondhand smoke, and smoke-free policies.^7^ Although the association between secondhand smoke exposure and COPD mortality has not been extensively examined, a study conducted in the Republic of Ireland demonstrated an immediate 38% reduction in COPD mortality following the implementation of a national smoke-free policy with the most predominant effects occurring among females and those aged ≥65 years.^17^ A follow-up study further determined that these all-cause and COPD-related mortality reductions were concentrated among those with the lowest socioeconomic status.^18^ Since active smoking prevalence did not appreciably change during this period, it was determined that the observed mortality reductions were attributable to decreased exposure to secondhand smoke. Further, these two studies were deemed to have a low risk of bias in a meta-analysis of 50 articles regarding smoke-free legislation and respiratory disorders.^19^

 Comprehensive smoke-free policies that prohibit smoking completely in public spaces are one public health countermeasure aimed at minimizing secondhand smoke exposure among non-smokers. Numerous studies have observed protective associations between the implementation of comprehensive smoke-free policies and COPD-related hospitalizations and mortality.^17,20-26^ Recently, a study found that county-level smoke-free policies reduce 30-day hospital readmissions due to COPD.^27^ In the United States, each state or county has the choice and responsibility for implementing smoke-free policies. As of April 1, 2020, 23 states had not adopted comprehensive smoke-free policies, leaving smoke-free policies to be determined at the county-level, and creating a wide variance in the implementation of these policies throughout the state.^28^

 The importance of determining the relationship between smoke-free policies and the risk of 30-day COPD mortality is multifaceted. For example, healthcare and public health professionals seek information about how to better protect the population’s health and save lives. At the policy level, it is paramount that enough evidence is available to support decision-making. In addition, 30-day COPD mortality rates are used as a hospital performance metric, prompting acute care providers and hospital administrators to seek opportunities to reduce COPD associated mortalities.^29^ Therefore, this study aims to investigate the relationship between smoke-free policies and 30-day mortality rates following COPD hospitalization in the United States.

## Methods

###  Data Source and Study Population

 The 2019 CMS Hospital Value-Based Purchasing (HVBP) Program dataset publicly reports aggregated hospital-specific 30-day risk-standardized mortality measures. The report includes condition-specific and procedure-specific mortality measures for inpatient discharges from July 1, 2015, to June 30, 2018. The five condition-specific measures are acute myocardial infarction, COPD, heart failure, pneumonia, and stroke; the one procedure-specific measure is coronary artery bypass graft. For this study, we utilized the CMS’s HVBP program to identify hospital COPD 30-day mortality rates throughout the United Stattes. Utilizing the 2017 County Federal Information Processing Standards (FIPS), we linked hospitals’ COPD mortality rates to the U.S. Tobacco Control Laws Database maintained by the American Nonsmokers’ Rights Foundation (ANRF).

 The ANRF was used to identify all state and county smoke-free policies from April 1936 to November 2018. The database reports on 1763 counties across all 50 states and the District of Columbia. ANRF collects policies through the review of public records including official websites, news services, and elected officials. A senior ANRF analyst indexes all reviewed policies and policy changes in accordance with established guidelines.^30^ Again utilizing the 2017 County FIPS, we linked hospitals to the counties in which they reside using the 2015-2018 American Hospital Association (AHA) Annual surveys, and the US Census Bureau Current Population Surveys. For this study, the AHA annual surveys were utilized to determine hospital census data and hospital characteristics, while the US Census Bureau Current Population Surveys were utilized to determine 1- and 5-year estimates for household adults ≥18 years of age.

###  Variables

 The study outcome was in-hospital 30-day mortalityfollowing admission for an acute exacerbation of COPD in patients ≥65 years of age enrolled in Medicare fee-for-service for the 12 months before admission. The diagnosis codes of COPD used by CMS were validated and reported by Lindenauer, Grosso, Wang, Wang, Krishnan, Lee, Au, Mularski, Bernheim, Drye^31^ using International Classification of Diseases, 9th Revision, Clinical Modification [ICD‐9‐CM] and revalidated by CMS QualityNet^32^ for ICD‐10‐CM. CMS risk-adjusted rates of 30-day mortality to determine a hospital’s case-mix (disease severity),^33^ and further calculate the rate for each hospital through fitting a hierarchical logistic regression to adjust for differences in hospital case mix index and to account for clustering of patients within a hospital. The measure calculates a 3-year average risk-standardized ratio as the number of COPD predicted deaths to the number of expected deaths, multiplied by the observed national mortality rate from July 1, 2015 to June 30, 2018.^34^ However, the measure does not adjust for socioeconomic status or race as outlined in National Quality Forum guidelines.

 The independent variable was county smoke-free law coverage. We based the variable on county or state smoke-free laws covering both public and private workplaces, restaurants, and bars through June 30, 2018. For this study, we excluded counties that implemented smoke-free policies during the study period (2015-2018) and we included the District of Columbia as a county. Consistent with the classification in prior literature, we defined 100% smoke-free as the implementation of a comprehensive smoke-free law prohibiting smoking indoors with no exceptions.^35-37^ We operationalized smoke-free law into three mutually exclusive categories: full coverage (FC = smoke-free law in workplaces, restaurants, and bars), partial coverage (PC = smoke-free in one to two locations), and no coverage (NC).

 To account for differences across counties, we included variables previously identified in the literature with the potential to impact respiratory outcomes in analyses. Prior studies have shown that there are sex-specific and race/ethnic-related differences in COPD manifestation,^38^ risks,^39^ and access to quality health services.^40^ Therefore, for the study period (2015-2018), we included a measure of the spatial distribution of multiple racial/ethnic groups simultaneously by calculating the average diversity score.^41^ We calculated the diversity score for each county using the sum of the log proportions of each of the 6 racial/ethnic groups represented in the United States, with a maximum value of 1.77 where equal proportions of all groups (33%) are present within the county.^41^ We also included the average total population covered by the smoke-free policy, percent of females ≥65 years of age, and population with ≥4 years college who are ≥65 years of age. In addition, we accounted for the percent of the county designated as a rural area, the average percent of the county living in poverty, and the average total number of hospitals within a county and counties within a state to account for disease burden.^42^

 Previous studies have shown that there are differences in hospital quality of care,^43^ safety performance,^44^ resource allocation,^45^ and community outreach^46,47^ based on hospital characteristics. Therefore, we included the average number of hospital beds within the study period (2015-2018), number of teaching hospitals, number of hospitals part of a system, percent of not-for-profit hospitals, and number of hospitals offering tobacco services as independent variables. We also included average Medicare and Medicaid percentages calculated by dividing total Medicare and Medicaid discharges by total inpatient admissions.^48^

###  Statistical Methods

 We designed the study to investigate the association between county-level smoke-free policies and average hospital COPD 30-day mortality rates within the study period. We based county inclusion on having at least one hospital present in the county that was participating in CMS’s HVBP program. Bivariate relationships were assessed between all hospital and county-level variables including mortality using *t* tests for dichotomous variables and analysis of variance for ordinal and continuous variables. Three hierarchical Poisson regression models were developed in accordance with previous work^27^ to estimate incidence rate ratios (IRRs) between county smoke-free law and hospital COPD mortality rate to account for nesting effects within states. The total count of individuals covered by the policy was included as an exposure term. The first partial model estimated county-level variables, the second partial model estimated hospital-level variables, and the third full model fitted both county- and hospital-level variables. All statistical testing was 2-sided, at a significance level of 0.05. We conducted all analyses using Stata version 14SE. Hierarchical models were fit using the MEPOISSON procedure in Stata.^49^ In accordance with the policy of the University, the research was categorized as exempt by the Institutional Review Board since the study analyzed secondary data that is publicly available.

## Results

 Of the 1171 counties included in this study period (2015-2018), 596 (51%) were FC, 385 (33%) were PC, and 190 (16%) were NC (Table 1). The NC counties tended to have a greater average COPD mortality (7.42 NC, 7.24 PC, 7.06 FC), greater percent of the county designated as rural (45.72 NC, 38.99 PC, 37.99 FC), higher average percent poverty (17.78 NC, 15.63 PC, 15.39 FC), higher average diversity index, ie, more heterogeneous population (0.71 NC, 0.62 PC, 0.60 FC), lower total population (49 864 NC, 76 169 PC, 85 014 FC), lower Medicaid population (0.20 NC, 0.22 PC, 0.26, FC), and lower percent of the population ≥65 with ≥4 years of college education (18.92 NC, 22.40 PC, 22.42 FC).

**Table 1:** Descriptive Statistics of Study Variables Between 2015-2018 by Smoke-Free Policy Category

**County Characteristics**	**No Coverage**		**Partial Coverage**		**Full Coverage**	
	**Mean (SD)**	**N**	**Mean (SD)**	**N**	**Mean (SD)**	**N**
Average diversity index	0.71 (0.25)	190	0.62 (0.27)	385	0.60 (0.29)	596
Total population covered by smoke policy	49 864 (91 380.51)	190	76 169 (198 926.50)	385	85 014 (365 260.50)	596
Average percentage of female 65+ years	55.80 (2.13)	190	55.34 (2.04)	385	55.37 (1.99)	596
Average percentage of population with 4+ years college	18.92 (8.24)	190	22.40 (10.08)	385	22.42 (8.68)	596
Percentage of county designated as rural	45.72 (27.50)	190	38.99 (27.44)	385	37.88 (27.77)	596
Average percentage poverty	17.78 (5.60)	190	15.63 (5.83)	385	15.39 (6.03)	596
Total number of hospitals	2.24 (3.26)	190	3.42 (5.51)	385	3.91 (7.09)	596
Total number of counties	59.93 (43.45)	190	52.88 (31.46)	385	53.03 (27.46)	596
COPD 30-day mortality	7.42 (3.09)	190	7.24 (2.94)	385	7.06 (2.88)	596
Average number of beds	315.84 (609.59)	190	591.67 (1289.22)	385	686.07 (1505.43)	596
Average total teaching in county	0.81 (1.68)	190	1.44 (2.81)	385	1.78 (3.72)	596
Average total system hospitals	2.21 (3.19)	190	3.39 (5.39)	385	3.88 (6.93)	596
Average percentage not-for-profit	46.48 (44.78)	190	59.91 (40.52)	385	68.59 (37.37)	596
Average total number of tobacco services offered by hospital	0.84 (1.29)	190	1.39 (2.02)	385	1.50 (2.43)	596
Average Medicare percentage	0.58 (0.14)	190	0.58 (0.14)	385	0.57 (0.15)	596
Average Medicaid percentage	0.20 (0.14)	190	0.22 (0.13)	385	0.26 (0.17)	596

Abbreviation: COPD, chronic obstructive pulmonary disease. Source: Author’s analysis of data (2015-2018) from the Centers for Medicare and Medicaid Services (CMS) Hospital Value-Based Purchasing (HVBP) Program. The County Federal Information Processing Standards (FIPS), the US Tobacco Control Laws Database maintained by the American Nonsmokers’ Rights Foundation (ANRF), American Hospital Association (AHA) Annual surveys, and the US Census Bureau Current Population Surveys (CPS).

 Concerning hospital characteristics, NC counties had fewer hospitals (2.24 NC, 3.42 PC, 3.91 FC), a lower number of beds (315.84 NC, 591.67 PC, 686.07, FC), fewer system hospitals (2.21 NC, 3.39 PC, 3.88 FC), fewer teaching hospitals (0.84 NC, 1.44 PC, 1.78 FC), a lower percentage of not-for-profit hospitals (46.48 NC, 59.91 PC, 68.59 FC), and fewer tobacco services offered by hospitals (0.84 NC, 1.39 PC, 1.50 FC).

 The hierarchical Poisson regression models (Table 2) revealed a consistent relationship between stronger smoke-free policies and a reduction in COPD mortality rates across all three models. Model 1 evaluated county characteristics associated with COPD mortality. Results showed that PC and FC counties experienced a reduced IRR of 0.80 (95% CI: 0.73, 0.89) and 0.75 (95% CI: 0.68, 0.83), respectively, compared to NC counties.

**Table 2:** Hierarchical Poisson Regressions of COPD Mortality Rate and County Characteristics

	**Model 1 (N = 1171)**	
	**IRR**	**95% CI**
Smoke-free policy (referent: NC)		
PC	0.80^a^	[0.73, 0.89]
FC	0.75^a^	[0.68, 0.83]
**County characteristics**		
Average diversity index	0.22^a^	[0.19, 0.25]
Percentage of county designated as rural	1.02^a^	[1.02, 1.02]
Average percentage of population ≥65 years with ≥4 years college	0.98^a^	[0.98, 0.98]
Average percentage of female ≥65 years	0.90^a^	[0.88, 0.91]
Average percentage poverty	1.02^a^	[1.01, 1.02]
Akaike information criterion	11 595.09	
Bayesian information criterion	11 640.68	

Abbreviations: COPD, chronic obstructive pulmonary disease; IRR, incidence rate ratio; NC, no coverage; PC, partial coverage; FC, full coverage. Source: Author’s analysis of data (2015-2018) from the Centers for Medicare and Medicaid Services (CMS) Hospital Value-Based Purchasing (HVBP) Program. The County Federal Information Processing Standards (FIPS), the U.S. Tobacco Control Laws Database maintained by the American Nonsmokers’ Rights Foundation (ANRF), American Hospital Association (AHA) Annual surveys, and the U.S. Census Bureau Current Population Surveys (CPS). *Notes*. IRR exponentiated coefficients; 95% CI in brackets; ^a^*P* <.001.

 Model 2 evaluated hospital characteristics and COPD mortality rates (Table 3). Our results associated PC and FC counties with reduced incidence rates of 0.86 (95% CI: 0.77, 0.94) and 0.71 (95% CI: 0.65, 0.79) compared to NC counties.

**Table 3:** Hierarchical Poisson Regressions of COPD Mortality Rate and Hospital Characteristics

	**Model 2 (N = 1171)**	
	**IRR**	**95% CI**
Smoke-free policy (referent: NC)		
PC	0.86^b^	[0.77, 0.94]
FC	0.71^a^	[0.65, 0.79]
**Hospital characteristics**		
Average number of hospital beds	0.99^a^	[0.99, 0.99]
Average Medicaid percentage	0.86	[0.69, 1.09]
Average Medicare percentage	1.35^b^	[1.08, 1.70]
Total number of hospitals part of a system	0.77^a^	[0.72, 0.82]
Total number of teaching hospitals	0.86^a^	[0.84, 0.88]
Average percentage of not-for-profit hospitals	1.00^a^	[1.00, 1.00]
Total number of hospital tobacco services	1.02	[0.99, 1.05]
Total number of hospitals	1.27^a^	[1.19, 1.35]
Akaike information criterion	12 582.9990	
Bayesian information criterion	12 643.7863	

Abbreviations: COPD, chronic obstructive pulmonary disease; IRR, incidence rate ratio; NC, no coverage; PC, partial coverage; FC, full coverage. Source: Author’s analysis of data (2015-2018) from the Centers for Medicare and Medicaid Services (CMS) Hospital Value-Based Purchasing (HVBP) Program. The County Federal Information Processing Standards (FIPS), the US Tobacco Control Laws Database maintained by the American Nonsmokers’ Rights Foundation (ANRF), American Hospital Association (AHA) Annual surveys, and the U.S. Census Bureau Current Population Surveys (CPS). *Notes*. IRR exponentiated coefficients; 95% CI in brackets; ^a^*P* <.001; ^b^*P* <.01.

 Model 3 evaluated both county and hospital characteristics associated with COPD mortality rates (Table 4). After adjusting for each (Model 3), only FC counties had a reduced rate of COPD mortality (IRR: 0.87, 95% CI: 0.79, 0.96) compared to NC counties. The percent of the county designated as rural (IRR: 1.02, 95% CI: 1.02, 1.02), average percent poverty (IRR: 1.03, 95% CI: 1.02, 1.03), total number of hospital tobacco services (IRR: 1.06, 95% CI: 1.03, 1.09) and total number of hospitals (IRR: 1.26, 95% CI: 1.18, 1.33) were all associated with increased risk of COPD mortality. The average percent population ≥65 years of age with ≥4 years of college (IRR 1.02, 95% CI: 1.02, 1.02), greater average percent female ≥65 years of age (IRR 0.97, 95% CI: 0.95, 0.99), greater average diversity index (IRR 0.54, 95% CI: 0.47, 0.62), greater average Medicaid percentage (IRR 0.42, 95% CI: 0.33, 0.52), greater average Medicare percentage (IRR: 0.63, 95% CI: 0.51, 0.79), greater total number of system hospitals (IRR: 0.80, 95% CI:0.75, 0.85), and greater number of teaching hospitals (IRR: 0.90, 95% CI: 0.88, 0.93) were all associated with decreases in COPD mortality rate.

**Table 4:** Hierarchical Poisson Regressions of COPD Mortality Rate and All County And Hospital Characteristics

	**Model 3 (N = 1171)**	
	**IRR**	**95% CI**
Smoke-free policy (referent: NC)		
PC	0.92	[0.83, 1.01]
FC	0.87^b^	[0.79, 0.96]
**County characteristics**		
Average diversity index	0.54^a^	[0.47, 0.62]
Percentage of county designated as rural	1.02^a^	[1.02, 1.02]
Average percentage of population ≥65 years with ≥4 years college	0.98^a^	[0.98, 0.98]
Average percentage of female ≥65 years	0.97^a^	[0.95, 0.99]
Average percentage poverty	1.03^a^	[1.02, 1.03]
**Hospital characteristics**		
Average number of hospital beds	0.99^a^	[0.99, 0.99]
Average Medicaid percentage	0.42^a^	[0.33, 0.52]
Average Medicare percentage	0.63^a^	[0.51, 0.79]
Total number of hospitals part of a system	0.80^a^	[0.75, 0.85]
Total number of teaching hospitals	0.90^a^	[0.88, 0.93]
Average percentage of not-for-profit hospitals	1.00^a^	[1.00, 1.00
Total number of hospital tobacco services	1.06^a^	[1.03, 1.09]
Total number of hospitals	1.26^a^	[1.18, 1.34]
Akaike information criterion	10 403.7704	
Bayesian information criterion	10 489.8858	

Abbreviations: COPD, chronic obstructive pulmonary disease; IRR, incidence rate ratio; NC, no coverage; PC, partial coverage; FC, full coverage. Source: Author’s analysis of data (2015-2018) from the Centers for Medicare and Medicaid Services (CMS) Hospital Value-Based Purchasing (HVBP) Program. The County Federal Information Processing Standards (FIPS), the US Tobacco Control Laws Database maintained by the American Nonsmokers’ Rights Foundation (ANRF), American Hospital Association (AHA) Annual surveys, and the U.S. Census Bureau Current Population Surveys (CPS). *Notes*. IRR exponentiated coefficients; 95% CI in brackets; ^a^*P* <.001; ^b^*P* <.01.

## Discussion

 Our findings show that comprehensive smoke-free policies are associated with a reduced risk of 30-day COPD mortality among hospitalized patients. Our data indicate that the risk of in-hospital mortality was the lowest for counties that had more extensive smoke-free policies than PC and even lower risk than NCs, indicating a dose-response relationship. Previous research on COPD re-admission rates and smoke-free policies in the United States reported a similar trend.^27^ For example, a 2020 quasi-experimental Brazilian-based study found that both partial and comprehensive smoke-free legislation was associated with a reduction in infant mortality.^50^ Another study that examined the association of smoke-free policies and incidence rates of cardiovascular disease showed that there is a long-term impact of these policies on lowering the risk of incident cardiovascular disease.^51^ Additionally, these findings are further supported by research conducted in Ireland,^17^ and Switzerland^52^ which found smoking bans to be associated with a reduction in mortality. Further, a systematic review of legislative smoking bans identified associations with reduced hospital admissions as well as reduced hospital mortality.^53^ As such, the current study’s findings provide incremental evidence that more comprehensive smoking bans within the United States are also associated with a reduction in COPD hospital mortality.

 Demographic and other study variables relating to the social determinants of health were important covariates in our models. Rurality, poverty rate, hospital tobacco services and the number of hospitals were each associated with worsened COPD outcomes, supporting findings from previous research.^45,54^ For example, a population-based study found an increased prevalence of COPD among older individuals and those with lower educational attainment, suggesting that these factors contribute to the onset of the disease which may explain the positive association indicated in this study.^55^ In contrast, counties with a greater percentage of older populations with higher educational attainment and a greater percentage of Medicare and Medicaid coverage were associated with a reduced risk of COPD mortality.

 Our findings also revealed that counties with a greater percentage of females were associated with lower COPD in-hospital mortality rates. This result also corresponds with past research indicating the association between counties with a greater percentage of females ≥65 years and decreased 30-day readmissions for COPD.^27^ However our findings as well as this previous study conflicts with the increasing trend of COPD mortality among women in the United States.^56^ In fact, previous COPD and gender studies have identified both an increased prevalence of COPD among women compared to men, as well as a higher mortality rate.^57,58^ Further, previous research shows that females are more likely to have reduced lung function and worse forms of COPD compared to males diagnosed with COPD.^59^ These findings support the importance of considering gender when addressing disparities in COPD outcomes, as well as a need to further investigate the causal mechanisms attributing to these gender differences.^59,60^

 Regarding hospital characteristics, we observed a relationship that the total number of system hospitals and teaching hospitals were associated with a decreased rate of 30-day COPD mortality. These findings are consistent with previous evidence indicating that system hospitals may have increased market power, translating into higher resource availability.^61^ Additionally, previous research among Medicare beneficiaries has shown that teaching hospital status has lower 30-day mortality rates for all hospitalizations when compared to non-teaching hospitals.^62^ Further, the association with both teaching and system hospitals suggest that counties with larger and integrated healthcare systems might be able to coordinate care more effectively for this patient population. Additionally, in this study, we observed greater COPD mortality for counties that had a greater number of hospitals. With respect to the number of hospitals, we could link this to counties with fragmented hospitals that are not part of a healthcare system and greater market competition.^61^

 In total, these results suggest that population-level interventions focused on more strict smoke-free policies as well as interventions aimed at reducing poverty, supporting education, and increased Medicare and Medicaid coverage may improve COPD mortality rates. This study is the first to investigate the association between county-level smoke-free policies and 30-day mortality following hospital admission for COPD. Strengths of the study include the extensive data sources allowing for precise estimates of the exposure and outcome as well as the ability to account for numerous potentially confounding factors, thus minimizing study bias. The multilevel hierarchical Poisson regression analysis ensured rigorous estimates of 30-day mortality risk by intrinsically addressing the nesting structure of the data, specifically counties within states and hospitals within counties.

 The limitations of this study include inability to adjust for potential confounding factors at the individual patient level and, in some cases, at the county level. Specifically, data limitations prevented adjustment for active or secondhand smoking prevalence, and air pollution measures as well as their corresponding changes over time. We were unable to adjust for air pollution due to inconsistent data availability at the county level for the years of the study. To address these limitations, we included the total population in each county covered by a smoke-free policy as an independent variable in statistical analyses. Moreover, prior evidence has shown a 2-fold hazard increase for hospitalization due to COPD exacerbations in winter compared to summer.^63^ However, in this study, we are unable to determine the seasonality of initial hospital admission or 30-day mortality or because the outcome measure for 30-day mortality is an overall hospital-level calculation.

## Conclusion

 The findings of this study are clear. Comprehensive, smoke-free policies with full coverage for workplaces, bars, and restaurants are associated with saving lives. It is also clear that partial smoke-free legislation is less protective than comprehensive legislation in reducing 30-day mortality following COPD hospital admission. For this reason, US counties and states should prioritize the implementation of smoke-free policies. Support for these policies benefits overarching public health programs, hospitals, and individual patients.

## Ethical issues

 In accordance with the policy of the University of North Florida the research was categorized as exempt by the University of North Florida’s Institutional Review Board since the study analyzed secondary data that is publicly available.

## Competing interests

 Authors declare that they have no competing interests.

## Authors’ contributions

 HH assisted in conceiving and designing the work, analyzing and interpreting the data, and writing part of the manuscript. SSS assisted in conceiving and designing the work, acquisition of data, literature search, writing part of the manuscript, and critical revision of manuscript for important intellectual content. EA assisted in writing part of the manuscript and critical revision of manuscript for important intellectual content. BP assisted in drafting part of the manuscript and literature search. AS assisted in conceiving and designing the work, interpreting the data, and drafting part of the manuscript.

## Funding

 The University of North Florida: Academic Affairs Faculty Scholarship Development Grant.
